# Flowering Time Modulation by a Vacuolar SNARE via *FLOWERING LOCUS C* in *Arabidopsis thaliana*

**DOI:** 10.1371/journal.pone.0042239

**Published:** 2012-07-27

**Authors:** Kazuo Ebine, Tomohiro Uemura, Akihiko Nakano, Takashi Ueda

**Affiliations:** 1 Department of Biological Sciences, Graduate School of Science, The University of Tokyo, Bunkyo-ku, Tokyo, Japan; 2 Molecular Membrane Biology Laboratory, RIKEN Advanced Science Institute, Wako, Saitama, Japan; 3 Japan Science and Technology Agency (JST), PRESTO, Honcho Kawaguchi, Saitama, Japan; Iwate University, Japan

## Abstract

The transition of plant growth from vegetative to reproductive phases is one of the most important and dramatic events during the plant life cycle. In *Arabidopsis thaliana*, flowering promotion involves at least four genetically defined regulatory pathways, including the photoperiod-dependent, vernalization-dependent, gibberellin-dependent, and autonomous promotion pathways. Among these regulatory pathways, the vernalization-dependent and autonomous pathways are integrated by the expression of *FLOWERING LOCUS C* (*FLC*), a negative regulator of flowering; however, the upstream regulation of this locus has not been fully understood. The *SYP22* gene encodes a vacuolar SNARE protein that acts in vacuolar and endocytic trafficking pathways. Loss of *SYP22* function was reported to lead to late flowering in *A. thaliana* plants, but the mechanism has remained completely unknown. In this study, we demonstrated that the late flowering phenotype of *syp22* was due to elevated expression of *FLC* caused by impairment of the autonomous pathway. In addition, we investigated the DOC1/BIG pathway, which is also suggested to regulate vacuolar/endosomal trafficking. We found that elevated levels of *FLC* transcripts accumulated in the *doc1-1* mutant, and that *syp22* phenotypes were exaggerated with a double *syp22 doc1-1* mutation. We further demonstrated that the elevated expression of *FLC* was suppressed by *ara6-1*, a mutation in the gene encoding plant-unique Rab GTPase involved in endosomal trafficking. Our results indicated that vacuolar and/or endocytic trafficking is involved in the *FLC* regulation of flowering time in *A. thaliana*.

## Introduction

Membrane trafficking is a major regulatory system for protein transport in eukaryotic cells. Evolutionary conserved regulatory molecules play substantial roles in regulating the budding of transport vesicles from organelles and the fusion to target membranes. For example, SAR/ARF GTPases regulate budding processes, and RAB GTPases and soluble N-ethyl-maleimide sensitive factor attachment protein receptors (SNAREs) are key regulators of membrane tethering and fusion. The SNARE proteins contain a heptad-repeat called the SNARE motif; according to sequence similarities in SNARE motifs, they are classified into three Q-SNARE groups and one R-SNARE group. In most cases, Q-SNAREs localize on target organelle membranes, and R-SNAREs localize on transport carriers. Typically, four SNARE molecules (or three, in the complex that contains a SNAP25-type SNARE) form a tight, stable complex that promotes membrane fusion between transport carriers and target organelles [1]. It is widely accepted that the combination of four SNARE proteins, in coordination with a particular RAB GTPase, confers specificity in membrane fusion.

SYP22/VAM3/SGR3 is a Q-SNARE that acts in vacuolar and endocytic transport pathways; this gene was originally identified by functional complementation of the yeast *vam3* mutant [2]. SYP22 localizes on the endosome/prevacuolar compartment (PVC) and the vacuolar membrane [2], [3], [4], [5]. There, SYP22 forms a complex with VTI11/ZIG, SYP5, and VAMP727 [6], [7], [8]. Loss-of-function *SYP22* mutants exhibit a wide spectrum of phenotypes, including wavy leaves, semi-dwarfism, immature leaf vascular tissues, excessive differentiation of idioblasts, and late flowering [9], [10], [11]. These phenotypes were synergistically enhanced by a second mutation in the cognate SNARE gene, *VAMP727*; conversely, the overexpression of *VAMP727* completely suppressed all *syp22-1* phenotypes [6]. Thus, *syp22-1* phenotypes appeared to result from defects in the endocytic and/or vacuolar transport pathways mediated by the SNARE complex of SYP22 and VAMP727, and not from defects in other unidentified SYP22 functions that are independent of membrane trafficking.

Most phenotypes of *syp22* mutants, like wavy leaves, semi-dwarfism, and abnormal leaf vascular tissues, are thought to be caused by a defect in polar auxin transport; the *syp22-4* mutation affects the polarized localization of PIN1, an efflux carrier of auxin, and the polar transport of auxin [10]. However, no studies have reported crosstalk between polar auxin transport and flowering regulation; this suggested that the late flowering phenotype of *syp22* could be due to a defect in an uncharacterized mechanism that linked flowering regulation to vacuolar/endocytic trafficking. In this study, we examined the mechanism underlying the late flowering phenotype of *syp22* to unveil this hidden link.

Upon floral induction, plants in the vegetative stage start producing floral meristems around the shoot apical meristem (SAM). In *A. thaliana*, the *FT* gene encodes an important floral inducer that is synthesized in leaves and transported to the SAM to induce expression of other floral inducers, including the *SUPPRESSOR OF OVEREXPRESSION OF CONSTANS1* (SOC1). Expression of *FT* and *SOC1* in *A. thaliana* is known to be regulated by at least four distinguishable genetic pathways: the photoperiodic-dependent, gibberellin-dependent, vernalization-dependent, and autonomous promotion pathways [12], [13]. The photoperiodic pathway modulates expression of *FT* in leaves in response to changes in the photoperiod [14]. The gibberellin pathway regulates flowering by controlling expression of *SOC1* in the SAM [15]. The vernalization and autonomous pathways cooperatively modulate expression of *FLC,* which represses the expression of *FT* and *SOC1* by directly interacting with the promoter regions of these genes [16]. Thus, *FLC* is a key integrator of the vernalization-dependent and autonomous promotion pathways.

To date, many putative nuclear factors have been identified that act in the autonomous pathway, including the RNA-binding or RNA-processing proteins, FCA, FPA, FLK, and FY, and chromatin modifiers, FVE and FLD [13]. However, no studies have reported the involvement of membrane trafficking in the regulation of *FLC* expression. Our results highlighted an unexpected link between the vacuolar SNARE and autonomous regulation of flowering in *A. thaliana*.

## Results

### *syp22-1* was Defective in Determination of Floral Meristem Identity

**Figure 1:**
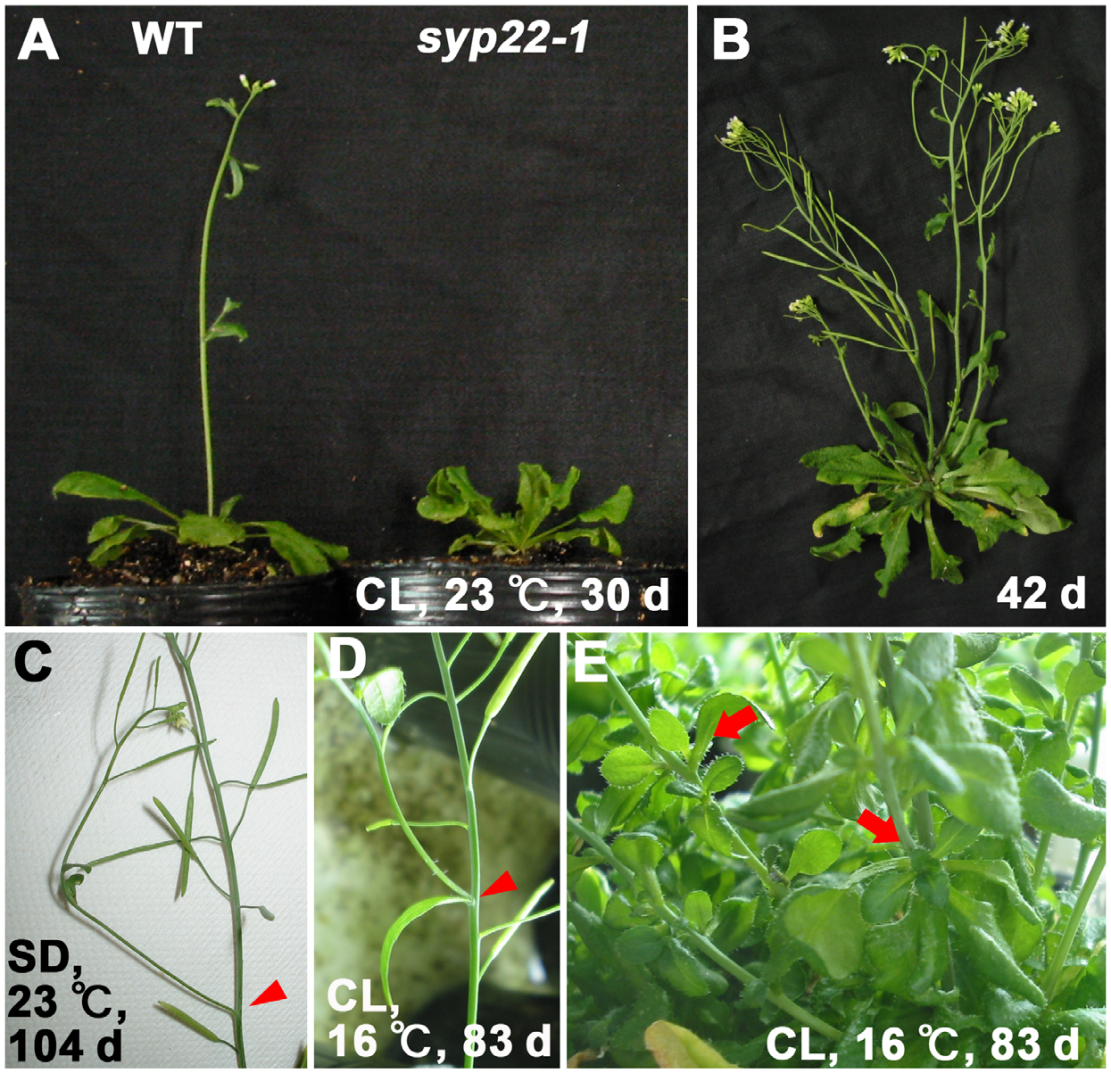
The *syp22-1* mutant plant showed abnormal floral meristem identity. (A) Wild-type (Columbia, left) and the *syp22-1* mutant (right) were grown in continuous light (CL) at 23°C for 30 days; (B) The *syp22-1* mutant is shown after 42 days. (C) *syp22-1* mutant grown under short-day conditions (SD). (D, E) Phenotypes of *syp22-1* grown under low temperature (16°C ) conditions. *syp22-1* exhibited conversion of floral meristems to inflorescence meristems under SD or low-temperature conditions (C, D, arrowheads). Aerial rosettes were also observed when grown under low-temperature condition (E, arrows).

Previously, *syp22-1* was reported to exhibit a broad range of phenotypes, including weak late flowering under continuous light (CL) (Fig. 1A) [9]. First, we investigated whether there was a functional link between *SYP22* and floral meristem identity determination. Under normal growth conditions (CL, 23°C), determination of the floral meristem identity was not affected in *syp22-1* (Fig. 1B). However, when grown under CL at 16°C, or under short-day conditions (SD: 8 h light and 16 h dark) at 23°C, the floral meristems of *syp22-1* were frequently converted to inflorescence meristems (Fig. 1C, D), and aerial rosettes were occasionally produced (Fig. 1E). A similar phenotype has been reported for other late flowering mutants, including *fld-2*, a mutant defective in the autonomous pathway [17].

**Figure 2:**
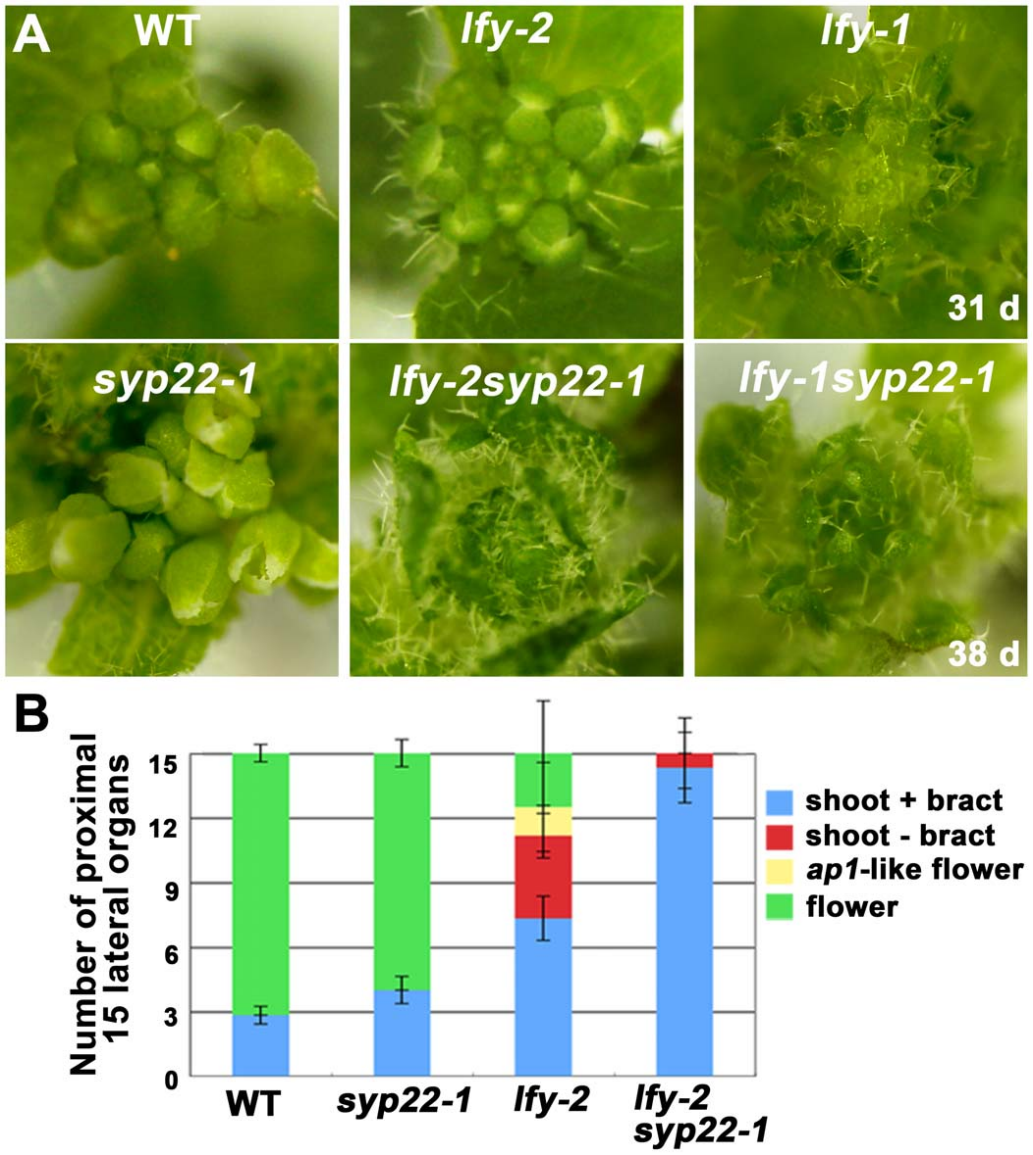
The *syp22-1* mutation synergistically aggravated the *lfy-2* mutation. (A) Morphology around shoot apical meristems of wild type (left, top) and mutant plants. The *lfy-2 syp22-1* double mutant (middle, bottom) exhibited a phenotype similar to that of *lfy-1* (right, top). (B) Numbers of proximal 15 lateral organs in wild type and mutants. Results are presented as the means ±S.D. (n = 12 plants). The *syp22-1* mutation alone did not markedly affect lateral organ identity, but it strongly promoted the transformation from flowers to inflorescences, when combined with the *lfy-2* mutation.

We then examined whether the abnormality in determination of floral meristem identity was related to the regulatory mechanism involving *LEAFY* (*LFY*), a key determinant of meristem identity. We generated double mutants between *syp22-1* and either a weak (*lfy-2*) or null (*lfy-1*) allele. The *syp22-1* mutation exaggerated the *lfy-2* phenotype, which suggested that the two genes operated in a synergistic manner; the double mutant *syp22-1 lfy-2* exhibited an indistinguishable phenotype from *lfy-1*, even when the double mutant plants were grown at 23°C in CL (Fig. 2A). On the other hand, the double mutant *syp22-1 lfy-1* exhibited the same phenotype as *lfy-1*. Similar synergistic enhancement of weak alleles of *lfy* mutations was reported in double mutants of *lfy* combined with other late flowering mutations, *ft, fd*, and *fld* [17], [18], [19].

We then analyzed the morphology of 15 proximal axillary organs that formed along the stems of wild-type and mutant plants. Compared with wild type and *syp22-1*, the *lfy-2* plants generated a larger number of lateral shoots, with or without a bract, instead of flowers. This was also synergistically enhanced in the double mutant, *syp22-1 lfy-2* (Fig. 2B, p<0.01, Student’s t-test). These results indicated that impairment of *SYP22* function caused a deleterious effect on the determination of meristem identity through a pathway that involved *LFY*.

### *syp22-1* Responded Normally to Gibberellin and Vernalization

**Figure 3:**
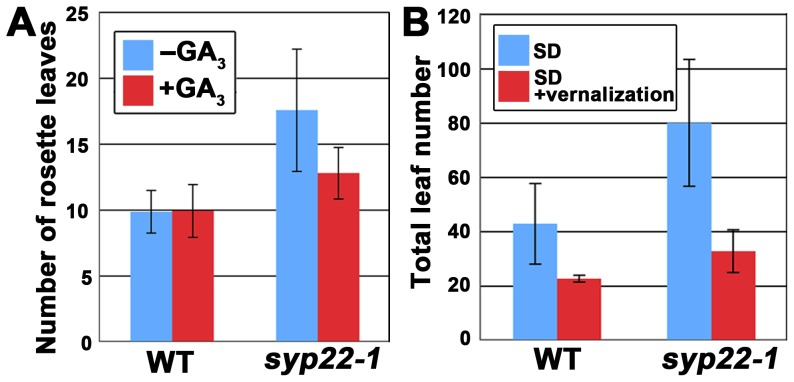
The *syp22-1* mutant responded normally to gibberellic acid, photoperiodic flowering induction, and vernalization treatment. (A) The number of rosette leaves in wild type (WT) and *syp22-1* mutants treated with (red) or without (blue) GA_3_. Results are presented as the means ±S.D. (n = 6 plants). (B) The total leaf numbers in wild type and *syp22-1* under SD with (red) or without (blue) vernalization. Results are presented as the means ±S.D. (n = 17 plants for SD and n = 10 plants for SD+ vernalization). Flowering of *syp22-1* was delayed in SD, which was suppressed by vernalization treatment (8 weeks at 4°C).

The four genetically distinguishable pathways that regulate the flowering time of *A. thaliana* are integrated by the expression of flowering pathway integrators, including the *LFY* gene [20]. To examine whether the *syp22-1* mutant was defective in these regulatory pathways, we first tested the *syp22-1* response to gibberellic acid (GA_3_). We found that GA_3_ treatment significantly promoted the flowering of *syp22-1* (Fig. 3A and Fig. S1A, p<0.01, Student’s t-test); this indicated that *syp22-1* retained responsiveness to gibberellin. Thus, the late flowering phenotype of *syp22-1* did not seem to be caused by impairment in the gibberellin-dependent pathway.

Next, we tested the *syp22-1* response to the photoperiod. The wild-type plants (Columbia accession) used in this study flowered after generating 12.1±1.1 leaves under CL conditions and after generating 42.7±14.8 leaves under SD. On the other hand, the *syp22-1* mutant generated a significantly larger number of leaves (79.8±23.4 leaves) when grown under SD than the wild type plants grown under SD (Fig. 3B and Fig. S1B, p<0.01, Student’s t-test). In contrast, the late flowering mutants defective in the photoperiod-dependent pathway are known to be insensitive to changes in photoperiod [21]; thus, the photoperiodic pathway did not seem to be markedly affected by the *syp22-1* mutation.

We then demonstrated that *syp22-1* responded normally to vernalization; vernalization treatment for 4 weeks at 4°C promoted flowering of *syp22-1* under SD (Fig. 3B, p<0.01, Student’s t-test). These results indicated that SYP22 was involved in the regulation of flowering time through regulatory pathways other than the above three pathways. Furthermore, we confirmed that the late flowering phenotype could be rescued by introducing the *GFP-SYP22* chimeric gene into the *syp22-1* mutant, under the regulation of its own promoter (Fig. S2); this confirmed that the late flowering phenotype was due to a loss of function in *SYP22*.

### Expression Level of *FLC* was Elevated in *syp22-1*

**Figure 4:**
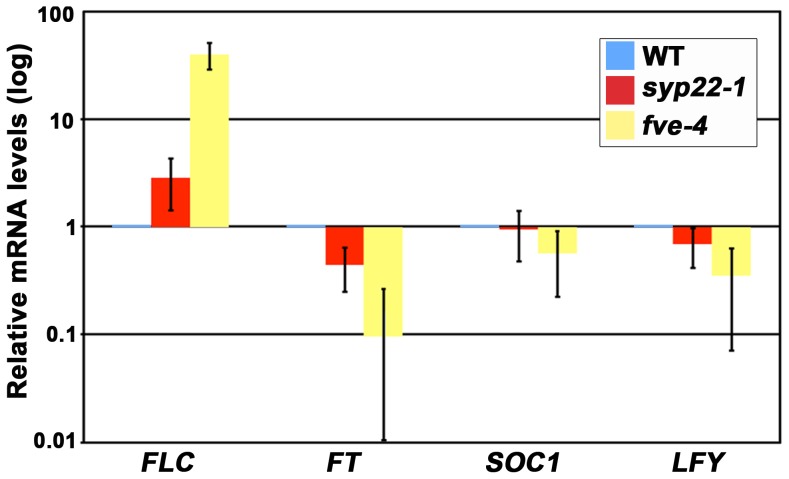
Expression level of *FLC* was elevated in *syp22-1* mutants. The expression levels of *FLC, FT, LFY,* and *SOC1* in 14-day-old wild type (WT), *syp22-1*, and *fve-4* seedlings were examined by qRT-PCR. In *syp22-1* plants, expression levels of *FLC* were elevated, which resulted in downregulation of downstream flowering pathway integrators. *fve-4*, an autonomous pathway mutant, was used as a control. Results are presented as means ±S.D. (n = 3–12).

**Figure 5:**
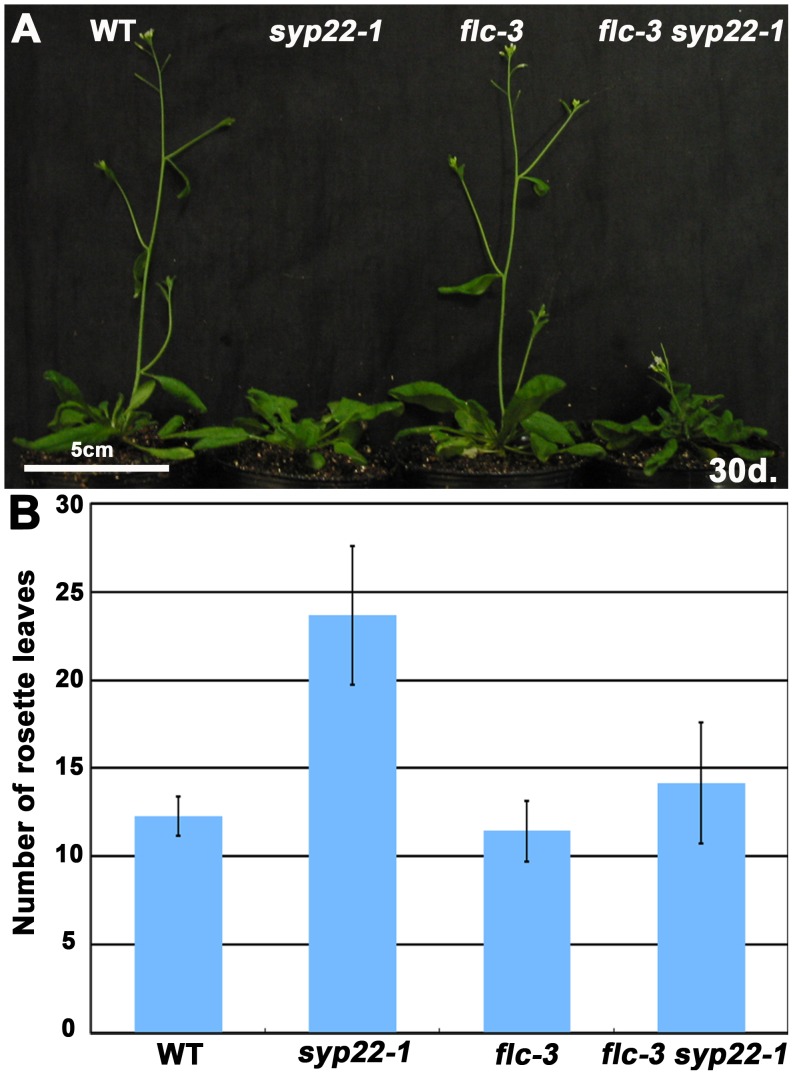
The late flowering phenotype of *syp22-1* was suppressed by *flc-3*. (A) Wild type (WT), *syp22-1, flc-3*, and *flc-3 syp22-1* plants were grown in CL for 30 days at 23°C. (B) Numbers of rosette leaves are shown for wild type (WT) and mutant plants grown under the same conditions as those in (A). Results are presented as means ±S.D. (n = 6 or 7 plants). The *flc-3* mutation suppressed only the late flowering phenotype of the *syp22-1* phenotypes.

The results mentioned above strongly suggested that the late flowering of *syp22-1* was caused by impairment in the last of a series of major regulatory pathways, the autonomous promotion pathway. *FLC* is a key regulator of the autonomous pathway. *FLC* encodes a MADS transcription factor that represses the expression of the floral pathway integrators, *FT*, *SOC1,* and *LFY* [13]. Thus, we reasoned that, if delayed flowering of s*yp22-1* was due to a defect in autonomous flowering promotion, then the mRNA level of *FLC* might be altered in this mutant. Accordingly, we examined the expression level of *FLC* and the floral pathway integrators in 14-day-old seedlings of the *syp22-1* mutant by quantitative RT-PCR (qRT-PCR). In *syp22-1*, *FLC* mRNA expression exceeded that of wild type by about three-fold (Fig. 4, p<0.01, Student’s t-test). Moreover, the mRNA expression of *FT* was significantly reduced compared to wild type (Fig. 4, p<0.01, Student’s t-test). In the *syp22-1* mutant transformed with *GFP-SYP22*, accumulation of mRNA for *FLC* was comparable to that in wild type (Fig. S2C). These results strongly suggested that the late flowering phenotype of *syp22-1* could be ascribed to elevated expression of *FLC*. We also confirmed this genetically; we reasoned that, if the elevated expression of *FLC* mRNA in *syp22-1* had directly caused its late flowering phenotype, then the elimination of *FLC* activity should suppress this phenotype. To test this, we generated a double mutant of *syp22-1* and *flc-3*, a null allele of *flc*, in a Columbia background. The endogenous expression of *FLC* was low in the Columbia accession, and *flc-3* exhibited a phenotype similar to wild type when grown in CL (Fig. 5) [22]. On the other hand, as expected, the *flc-3 syp22-1* double mutant generated a significantly smaller number of leaves (14.2±3.4 leaves) before flowering than *syp22-1* (23.7±3.9 leaves, p<0.01, Student’s t-test) under CL conditions; thus, the double mutant was comparable to the wild type (12.3±1.1 leaves) and *flc-3* (11.4±1.7 leaves) plants (Fig. 5). These results clearly demonstrated that the elevated level of *FLC* mRNA expression conferred a late flowering phenotype on *syp22-1*. Among the pleiotropic phenotypes of *syp22-1*, the *flc-3* mutation suppressed only the late flowering phenotype; in contrast, other phenotypes, like wavy leaves and semi-dwarfism, were not remedied in the *flc-3 syp22-1* double mutant (Fig. 5A). This indicated that the late flowering phenotype of *syp22-1* was conferred by a mechanism distinct from that responsible for other phenotypes, which are most likely due to impairments in polar auxin transport.

### Mutations in BIG, a Putative Regulator of Membrane Trafficking, and ARA6 also Altered *FLC* Expression

**Figure 6:**
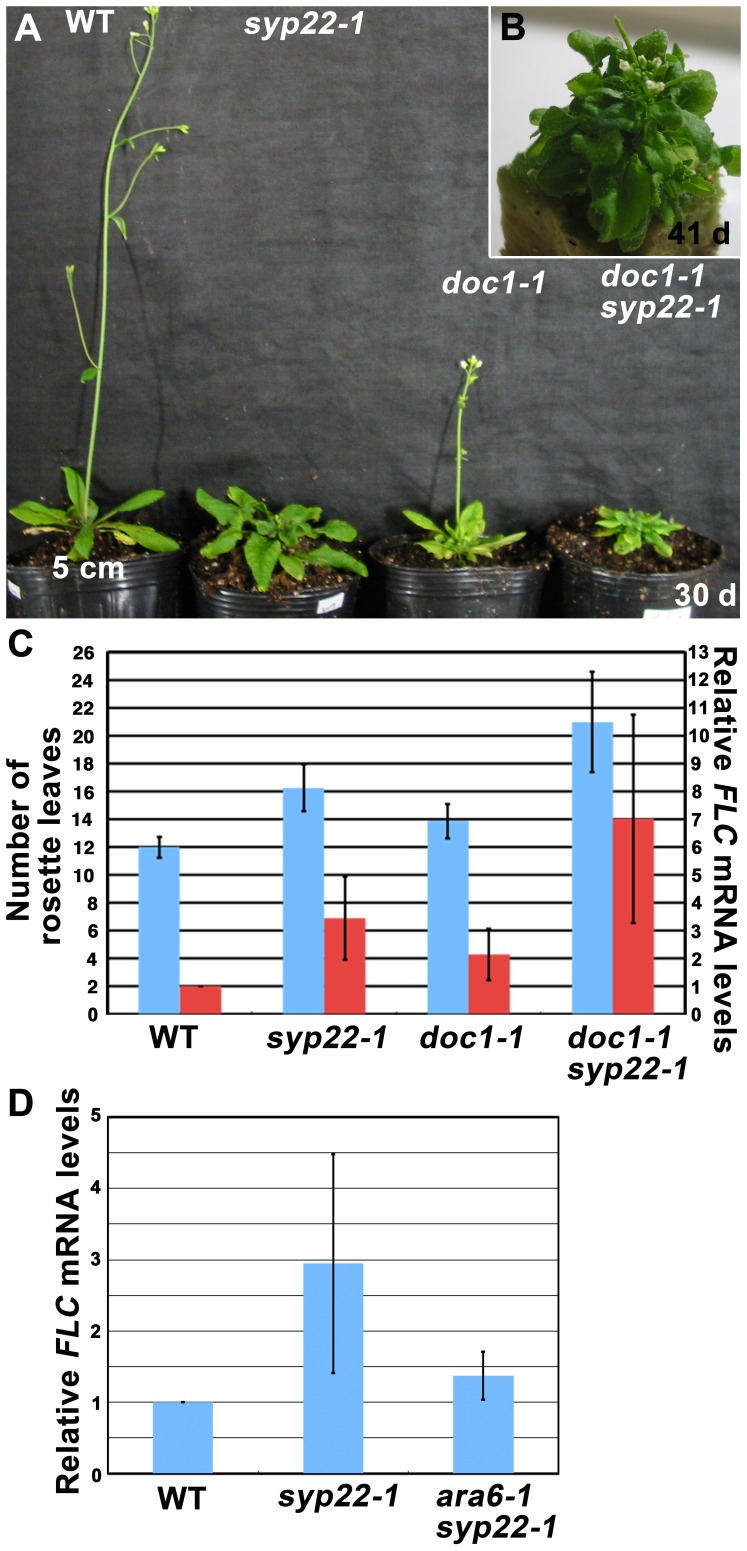
Effect of *doc1-1* and *ara6-1* mutations on expression of *FLC* in *syp22-1*. (A) Wild type (WT), *syp22-1, doc1-1*, and *doc1-1 syp22-1* plants were grown in CL for 30 days at 23°C. (B) *doc1-1 syp22-1* plants grown in CL for 41 days at 23°C. (C) Numbers of rosette leaves (blue) and expression levels of *FLC* (red) are shown for wild type (WT), *syp22-1, doc1-1*, and *doc1-1syp22-1* plants grown in CL at 23°C. Results are presented as means ±S.D. (n = 8 plants, 3 experiments of qRT-PCR). Results of qRT-PCR of *FLC* were normalized by the expression of *TUA3*. (D) The elevated expression level of *FLC* in *syp22-1* was suppressed by the *ara6-1* mutation. Expression levels of *FLC* in 14-day-old wild type (WT), *syp22-1*, and *ara6-1syp22-1* seedlings were examined by qRT-PCR. Results are presented as means ±S.D. (n = 5 experiments).

The results described above strongly suggested that vacuolar and/or endocytic trafficking pathways were involved in autonomous regulation of flowering. We then examined whether other membrane trafficking mutants exhibited a similar phenotype. We tested whether a genetic mutant of *BIG*, which encodes a Calossin-like protein, might also exhibit elevated *FLC* expression. *BIG* was originally identified as the gene responsible for the *doc1* mutation [23]. BIG was also reported to be required for the auxin-mediated inhibition of endocytosis [24]. Furthermore, treatment with naphthylphthalamic acid (NPA), an inhibitor of polar auxin transport, altered subcellular localization of PIN1 in the *doc1* mutant [23]. Based on these results, mutations in *BIG* were implicated in impairments of membrane trafficking pathways, including the endocytic and/or vacuolar transport pathways. Intriguingly, some mutant alleles of *BIG* have been also reported to exhibit the late flowering phenotype [25], [26]. These results implied that *BIG* was also involved in regulation of *FLC* expression. Among the *big* mutations, the allele that caused the most severe late flowering phenotype was *doc1-1* [26]. Under our CL conditions, *doc1-1* exhibited a late flowering phenotype (13.9±1.2 leaves, Fig. 6A, C) compared with wild type (12.0±0.8 leaves); this phenotype was similar to that of *syp22-1* (16.3±1.7 leaves, Fig. 6A, C). We then generated a *doc1-1 syp22-1* double mutant, which exhibited a more severe late flowering phenotype (21.0±3.6 leaves, Fig. 6A–C, p<0.01, Student’s t-test). A qRT-PCR analysis also indicated that the level of *FLC* mRNA expression was slightly increased in *doc1-1*, and this was enhanced in the double mutant, *doc1-1 syp22-1* (Fig. 6C). Thus, BIG and SYP22 could be involved in the same regulatory pathway for *FLC* expression.

We then examined the effect of a mutation in the other membrane trafficking regulator, ARA6, which is a plant-unique Rab GTPase involved in trafficking between multivesicular endosomes and the plasma membrane [27]. We have already reported that the *ara6-1* mutation suppressed the late flowering phenotype of *syp22-1*. We examined whether the elevated expression level of *FLC* in *syp22-1* is also suppressed by *ara6-1*, and found that *FLC* expression was significantly reduced in *ara6-1 syp22-1* (Fig. 6D, p<0.05, Student’s t-test). This result also supports a possible link between membrane trafficking and flowering regulation.

## Discussion

The *syp22* mutants have exhibited pleiotropic phenotypes, including late flowering, semi-dwarfism, waved leaves, immature leaf vascular tissues, and excessive idioblast differentiation. All these phenotypes, except late flowering, have been thought to result from a misdistribution of the phytohormone, auxin [9], [10], [11]. In contrast, the reason that mutations in *SYP22* engendered late flowering remained unclear. In the present study, we demonstrated that elevated expression of *FLC* in the *syp22-1* mutant was a direct cause of the late flowering phenotype (Figs. 4 and 5). The *syp22-1* mutant was capable of responding to vernalization treatment (Fig. 3D), which suggested that the *syp22-1* mutation resulted in impairment in the autonomous promotion pathway. We further demonstrated that the expression level of *FLC* was elevated in another membrane trafficking mutant, *doc1-1* (Fig. 6A–C), and that *ara6-1,* a mutation in the gene encoding a plant-unique Rab GTPase, suppressed elevated expression of *FLC* in *syp22-1* (Fig. 6D). These results indicated that endocytic and/or vacuolar trafficking pathways were involved in the autonomous promotion of flowering in the Columbia accession of *A. thaliana*.

Our findings clearly indicated that the vacuolar SNARE, SYP22, was required for proper regulation of *FLC*, a key regulator of flowering time. This result may elucidate the recent finding that *TERMINAL FLOWER 1* (*TFL1*), a homolog of *FT*, which is required for repression of the transition from the inflorescent meristem to the floral meristem, played a critical role in membrane trafficking to protein storage vacuoles (PSVs) [28]. Based on that finding, the authors proposed a novel function of the PSV: the storage of factors necessary for flowering and meristem maintenance. Other genetic studies indicated that *TFL1* acted downstream of *FVE* and *FCA*, which are upstream regulators of *FLC* in the autonomous pathway [19], [29]. In addition, *tfl1-1*, a loss-of-function mutant of *TFL1*, exhibited an early flowering phenotype and reduced expression of *FLC* [30]. Those results suggest that *TFL1* may also be involved in autonomous promotion of flowering. In our previous study, we found that the *syp22* mutant also exhibited defects in the biogenesis of PSVs in *A. thaliana* [6]. Taken together, these results suggest the hypothesis that *syp22-1* is defective in transport or storage of the flowering factors harbored in PSVs that affect autonomous promotion of flowering via modulation of *FLC* expression. Our present results clearly indicated that the flowering phenotype of *syp22-1* was caused by a mechanism genetically separable from that responsible for other phenotypes, like semi-dwarfism and wavy leaves, which are most likely due to abnormal polar auxin transport. However, the predicted flowering regulators could be transported via the same trafficking pathway as the efflux carriers of auxin, because mutants of both *SYP22* and *BIG*/*DOC1* also exhibited abnormalities in polarized localization of the PIN1 protein [10], [23].

Another potential hypothesis is that SYP22 could be involved in transcriptional regulation of *FLC* via chromatin remodeling. In mammalian cells, an adaptor protein containing PH domain, PTB domain, and leucine zipper motif 1 (APPL1) has been identified as a RAB5 effector, which transmits a signal from the endosomes to the nucleus [31]. APPL1 was also shown to interact with the nucleosome remodeling and deacetylase (NuRD) complex to regulate histone deacetylase (HDAC) 2 activity [32]. The level of *FLC* expression is tightly regulated, and this depends on the extent of chromatin modification; moreover, FVE and FLD were demonstrated to be components of the HDAC complex responsible for deacetylation of the *FLC* locus [33], [34]. If similar regulatory mechanisms of endosomal signaling followed by chromatin modification are conserved in plants, the mutation in *SYP22* or *BIG* could affect these machineries, which could lead to elevation of *FLC* expression. Further study of molecular components that mediate endosomal signaling in plant cells would be required to investigate these hypotheses.

In conclusion, this study revealed a linkage between the fundamental cellular function of membrane trafficking and the higher ordered regulation of plant flowering. Future study of flowering from the viewpoint of organelle functions that are associated with membrane trafficking would elucidate our understanding of this regulatory system.

## Materials and Methods

### Plant Materials and Growth Conditions

*A. thaliana* (Columbia accession) seeds were sown on MS plates after sterilization. After a 2-day incubation at 4°C, plants were grown at 23°C under CL or SD (8 h light, 16 h dark). After 14 days (CL) or 21 days (SD) of incubation on plates, plants were transferred to the soil. For the vernalization treatment, plants were grown for 3 weeks at 23°C in SD, then incubated at 4°C for 8 weeks in SD.

The *lfy-1* and *lfy-2* mutants were obtained from ABRC; the *fve-4* [33] and *flc-3* mutants [22] were kindly provided by T. Araki (Kyoto Univ.). The *doc1-1* mutant [35] was a kind gift from S. Sawa (Univ. of Tokyo). *syp22-1/vam3-t* and *ara6-1syp22-1* were described elsewhere [27].

### GA_3_ Treatment

Seeds were sown on MS plates and cultured for 14 days under CL conditions. Plants were transferred to the soil, and then sprayed with 20 µM GA_3_ (Wako) or water twice per week.

### qRT-PCR

RNA was extracted from the aerial parts of 14-day-old plants with the RNAqueous-4 PCR kit (Ambion). Superscript III (Invitrogen) was used for reverse transcription. qRT-PCR was performed on a Light cycler 480 system (Roche) with specific sets of primers, including *FLC*: FLCf. 5′-gactgccctctccgtgacta-3′ and FLCr. 5′-ttctcaacaagcttcaacatgag-3′; *FT*: FTf. 5′-ggtggtgaagacctcaggaa-3′ and FTr. 5′-ggttgctaggacttggaacatc-3′; *SOC1*: SOC1f. 5′-aacaactcgaagcttctaaacgtaa-3′ and SOC1r. 5′-cctcgattgagcatgttcct-3′; *LFY*: LFYf. 5′-ttgatgctctctcccaagaag-3′ and LFYr. 5′-ttgacctgcgtcccagtaa-3′; *TUA3*: TUA3f. 5′-aagaagtctaagcttggttttaccat-3′ and TUA3r. 5′-agggaatgcgttgagagaac-3′. Primers and probes were selected according to the Universal Probe Library Assay Design Center (Roche, https://www.roche-applied-science.com/sis/rtpcr/upl/index.jsp?id=uplct_030000).

### Accession Numbers

The Arabidopsis Genome Initiative locus identifiers for the genes mentioned in this article are: At5g46860 (*SYP22/VAM3/SGR3*), At5g61850 (*LFY*), At1g65480 (*FT*), At2g45660 (*SOC1*), At5g10140 (*FLC*), At2g19520 (*FVE*), At3g54840 (ARA6), and At3g02260 (*BIG/DOC1*).

## Supporting Information

Figure S1 **The** ***syp22-1*** **mutant responded normally to gibberellic acid and photoperiodic flowering induction.** (A) The *syp22-1* mutant was grown in CL for 30 days at 23°C, with (right) or without (left) GA_3_ treatment. (B) Wild-type (left) and *syp22-1* mutant (right) grown under short-day conditions (SD, 8 h light/16 h dark) for 65 days at 23°C.
(TIF)

Figure S2 **Phenotypes of** ***syp22-1*** **were rescued by expressing GFP-SYP22.** (A) Wild type (WT), *syp22-1* (-), and two independent transgenic *syp22-1* lines (#3-1 and #5-2) rescued with GFP-SYP22 expression under the regulation of the authentic promoter (*proSYP22::GFP-SYP22*) were grown under SD for 60 days at 23°C. (B) Numbers of rosette leaves are shown for wild type (WT), *syp22-1*, and *syp22-1* rescued with GFP-SYP22 expression. Results are presented as means ±S.D. (n = 6 plants). (C) The expression levels of *FLC* in wild type (WT), *syp22-1*, and *syp22-1* rescued with GFP-*SYP22* were examined by qRT-PCR. Results of qRT-PCR of *FLC* were normalized by the expression of *TUA3*. Results are presented as means ±S.D. (n = 3).
(TIF)
