# Supplementary figures and images for: Flowering Time Modulation by a Vacuolar SNARE via FLOWERING LOCUS C in Arabidopsis thaliana
Source: PLoS One. 2012 Jul 27;7(7):e42239. doi: 10.1371/journal.pone.0042239 (PMC3407077; doi:10.1371/journal.pone.0042239)

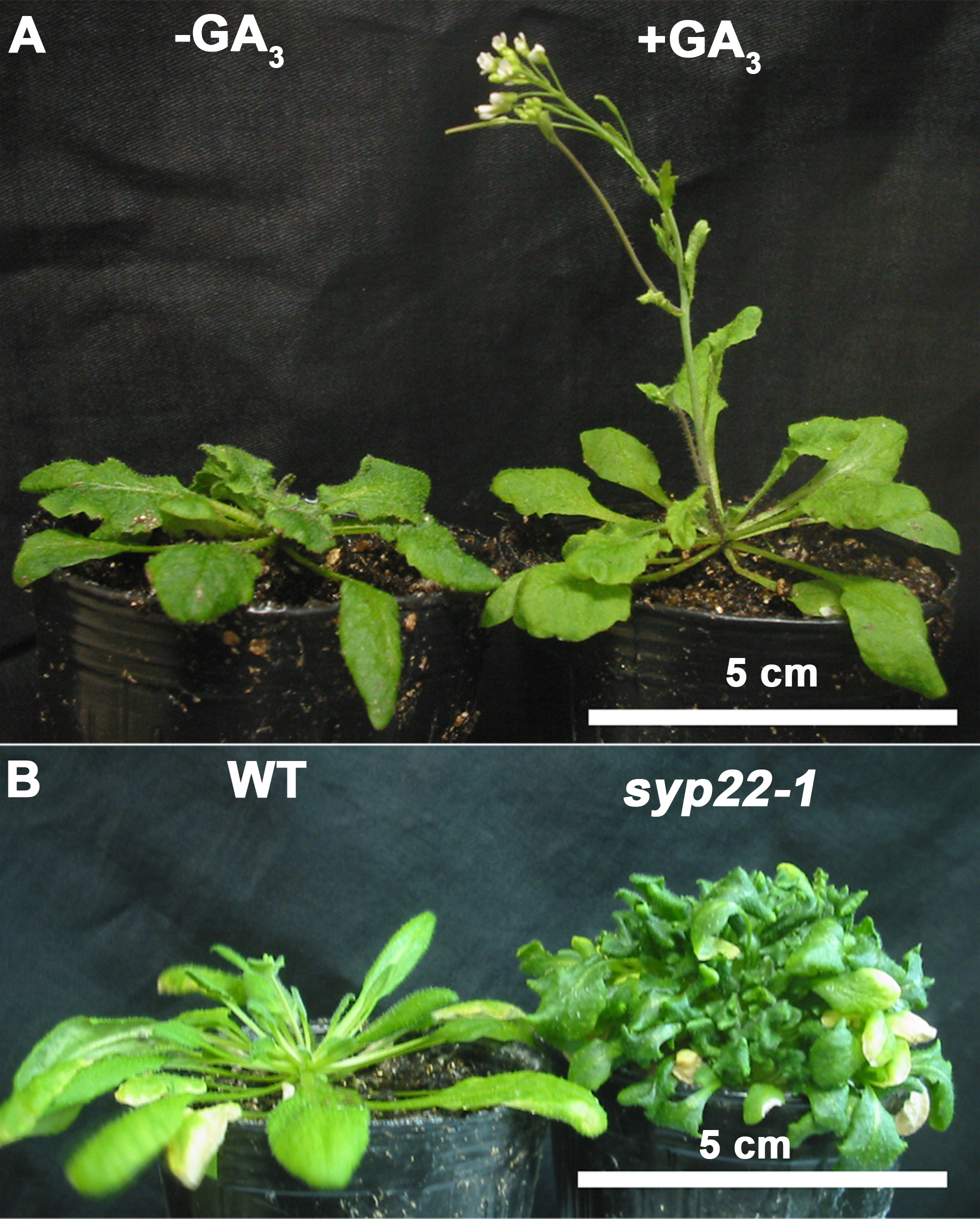

Supplement: Figure S1 — The syp22-1 mutant responded normally to gibberellic acid and photoperiodic flowering induction. (A) The syp22-1 mutant was grown in CL for 30 days at 23°C, with (right) or without (left) GA3 treatment. (B) Wild-type (left) and syp22-1 mutant (right) grown under short-day conditions (SD, 8 h light/16 h dark) for 65 days at 23°C. (TIF) [file pone.0042239.s001.tif]

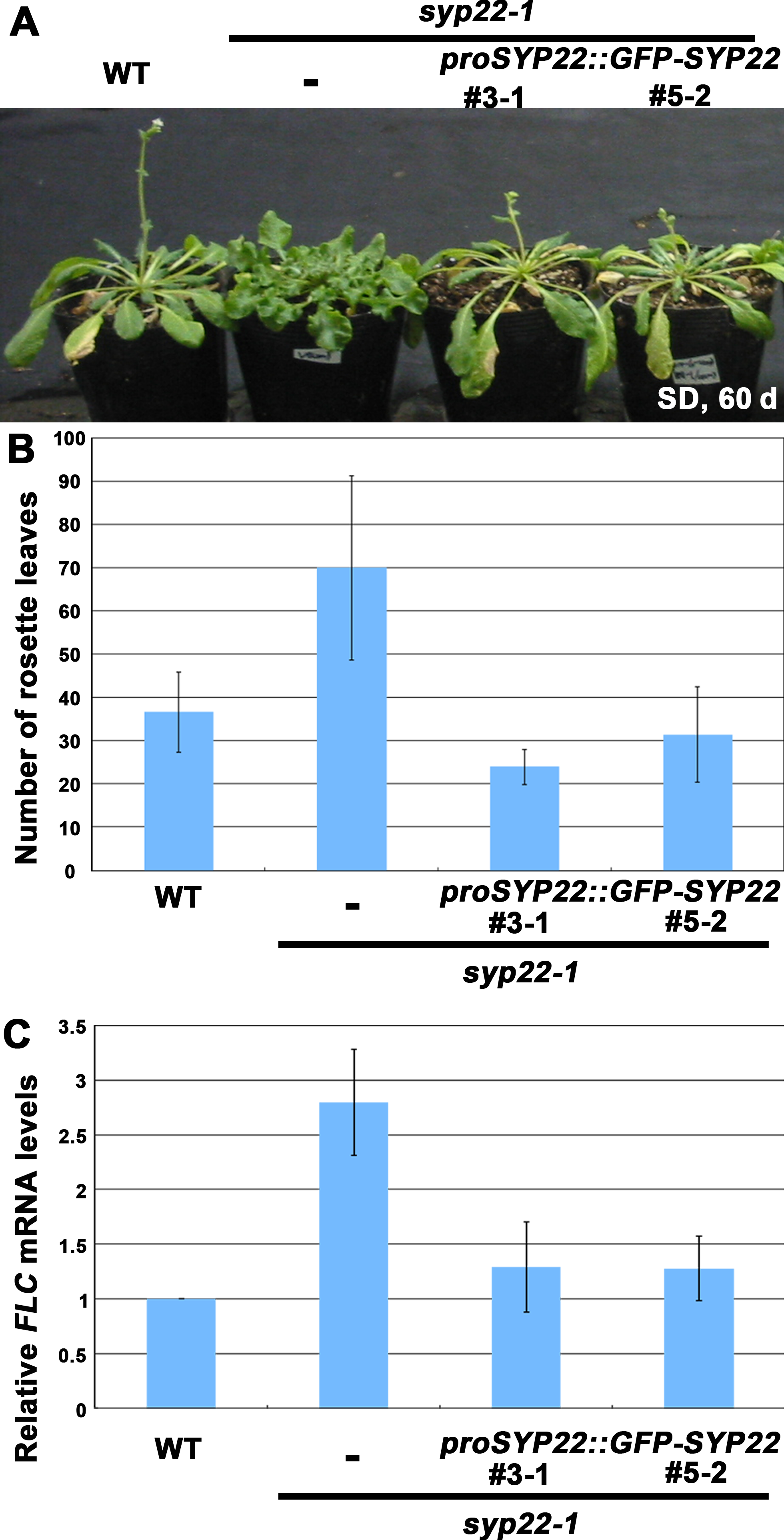

Supplement: Figure S2 — Phenotypes of syp22-1 were rescued by expressing GFP-SYP22. (A) Wild type (WT), syp22-1 (-), and two independent transgenic syp22-1 lines (#3-1 and #5-2) rescued with GFP-SYP22 expression under the regulation of the authentic promoter (proSYP22::GFP-SYP22) were grown under SD for 60 days at 23°C. (B) Numbers of rosette leaves are shown for wild type (WT), syp22-1, and syp22-1 rescued with GFP-SYP22 expression. Results are presented as means ±S.D. (n = 6 plants). (C) The expression levels of FLC in wild type (WT), syp22-1, and syp22-1 rescued with GFP-SYP22 were examined by qRT-PCR. Results of qRT-PCR of FLC were normalized by the expression of TUA3. Results are presented as means ±S.D. (n = 3). (TIF) [file pone.0042239.s002.tif]
